# Multifocal Oral Epstein–Barr Virus-Positive Mucocutaneous Ulcers Associated with Dual Methotrexate and Leflunomide Therapy: A Case Report

**DOI:** 10.1055/s-0041-1739545

**Published:** 2022-01-11

**Authors:** Sumana Kunmongkolwut, Chatchawan Amornkarnjanawat, Ekarat Phattarataratip

**Affiliations:** 1 Department of Oral Pathology, Faculty of Dentistry, Chulalongkorn University, Bangkok, Thailand; 2 Department of Dentistry, Surin Hospital, Surin, Thailand

**Keywords:** Epstein–Barr virus infections, leflunomide, lymphoproliferative disorders, methotrexate, oral ulcerations

## Abstract

Epstein–Barr virus (EBV)-positive mucocutaneous ulcer (EBVMCU) is a unique clinicopathologic entity of lymphoproliferative disorder, occurring in immunosuppressed patients. Due to its rarity, EBVMCU may be under-recognized by clinicians as well as pathologists. In addition, its clinical and histopathologic features overlap with other benign and malignant conditions, making a diagnosis challenging. This report presents an unusual case of multifocal oral EBVMCUs in a 52-year-old female patient with rheumatoid arthritis, receiving the combination of methotrexate and leflunomide for 5 years. The patient presented with persistent multiple large painful ulcers involving her palate and gingiva for 6 months. The histopathologic examination revealed extensive ulceration with diffuse polymorphic inflammatory infiltrate admixed with scattered atypical lymphoid cells showing occasional Hodgkin and Reed/Sternberg-like cell features. These atypical cells showed immunoreactivity for CD20, CD30 and MUM1/IRF4. EBV-encoded small RNA in situ hybridization was positive, validating the presence of EBV-infected cells. Two months after discontinuation of both immunosuppressive medications, oral lesions gradually regressed. At 9-month follow-up, no evidence of relapsing oral EBVMCU has been observed. The multifocal presentation of EBVMCU is rare and could be resulted from the overwhelming immune suppression by long-term use of dual immunosuppressants. Its diagnosis requires comprehensive correlation of patient history, clinical findings, histopathologic, and immunophenotypic features. The ability of EBVMCU to regress following removal of immunosuppressive causes is in drastic contrast to a variety of its potential clinical and histopathologic mimics. Therefore, accurate diagnosis is crucial to avoid unnecessary patient management and achieve optimal patient outcomes.

## Introduction

Epstein–Barr virus-positive mucocutaneous ulcer (EBVMCU) is a distinct B cell lymphoproliferative disorder (LPD), first described by Dojcinov et al in 2010. 1 Since then, additional cases were reported, and EBVMCU was coined as a new disease entity by the World Health Organization (WHO) in 2017. 2 EBVMCU commonly occurs in older adults with a median age of 71 years (range: 0.4–101 years), and shows a slight female predilection with the male-to-female ratio of 0.82 to 1. 3 EBVMCU typically appears as a solitary, sharply demarcated ulcer, most often arising in the oral mucosa (gingiva, tongue, lip, buccal mucosa, and palate) and oropharynx, followed by gastrointestinal tract mucosa, and skin. 3 Multifocal lesions are rare. EBVMCU patients often experience various forms of immunosuppression, including iatrogenic immunosuppression (e.g., solid organ or bone marrow transplant, autoimmune diseases), age-related immunosenescence, or less frequently primary immunodeficiencies and human immunodeficiency virus (HIV)/acquired immunodeficiency syndrome (AIDS). 3 4 5 The etiopathogenesis of EBVMCU are related to the failure of host–virus homeostasis, resulting in the consuming EBV infection. In patients with advanced age, there is a reduced capacity to generate new naïve T cells, owing to a markedly restricted epitope-specific T cell repertoire. This leads to the accumulation of clonal or oligoclonal restricted CD8+ T cells with reduced functionality. 6 The decline in T cell-mediated immune surveillance results in the expansion and transformation of EBV-infected B cell reservoirs. 1 5

Herein, we report a rare case of multifocal EBVMCUs involving the gingival and palatal mucosa of a rheumatoid arthritis (RA) patient using the combination of methotrexate (MTX) and leflunomide therapy.

## Case Report

A 52-year-old female presented with multiple persistent painful ulcers involving the right posterior hard palate and gingiva for 6 months. Her medical history included RA, and she had been treated with the combination of low-dose MTX (7.5 mg twice weekly) and leflunomide (20 mg daily) for 5 years, 3 months and 4 years, 10 months, respectively. She occasionally took naproxen to relieve pain and omeprazole together with simethicone to mitigate gastrointestinal symptoms. Her other medications included folic acid, ferrous fumarate, and multivitamins for anemia of inflammation as well as hydroxyzine, cetirizine, and topical urea cream for RA-related skin conditions. She denied smoking or drinking habits. Upon physical examination, neither regional nor systemic lymphadenopathy was evident.

**Fig. 1:**
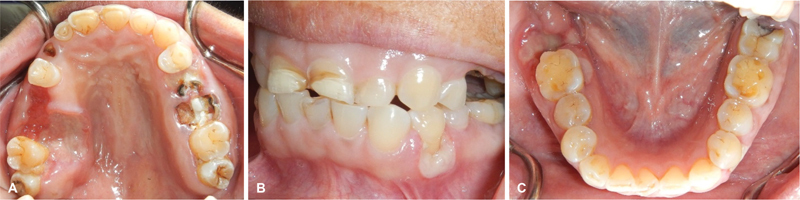
Clinical presentation of oral Epstein–Barr virus-positive mucocutaneous ulcer. ( **A** ) A sharply demarcated, deep, irregular ulcer at the right posterior palate with erosive area at the edentulous ridge of right maxillary second premolar and first molar. Similar ulcers are also noted at ( **B** ) buccal gingiva of the left mandibular first premolar ( **C** ) lingual gingiva of right mandibular first molar as well as the mucosa around post-extraction socket of the second molar.

**Fig. 2:**
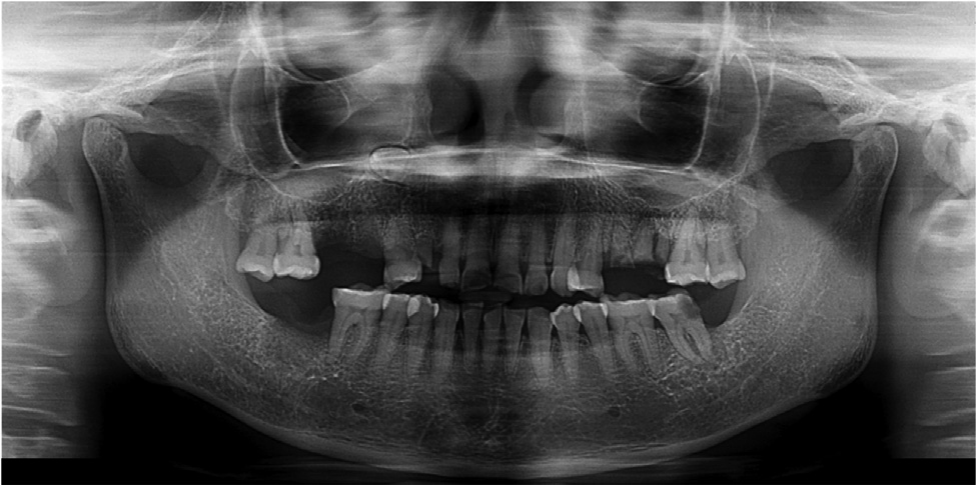
Panoramic radiograph showing unremarkable underlying bone involvement beneath the ulcers.

Intraoral examination revealed multiple, sharply demarcated, deep ulcers involving edentulous areas of the right maxillary second premolar and first molar extending to the right posterior hard palate, the gingiva surrounding the grossly carious left maxillary second premolar ( Fig. 1A ), the attached gingiva buccal to the left mandibular first premolar ( Fig. 1B ), and lingual to the right mandibular first molars, and the edentulous right mandibular second molar area ( Fig. 1C ). These ulcers varied in size with the largest diameter measuring 2.5 cm and were covered by yellowish white pseudomembranes. Panoramic radiograph revealed no significant bone involvement by the lesions ( Fig. 2 ).

Additional laboratory studies were performed. Complete blood count revealed a decrease in the number of red blood cells (3.7×10 ^6^ /µL), hemoglobin (10.7 g/dL), and hematocrit (33.4%), indicating mild anemia. Peripheral blood smear revealed normochromic, normocytic red blood cells. In addition, a normal white cell count was observed. The white cell differential showed an increase in the number of monocytes (11.3% of white blood cell count; absolute monocyte count = 0.6 × 10 ^3^ /µL). A slight increase in aspartate aminotransferase (37 U/L) and globulin (4.1 g/dL) and mildly decreased estimated glomerular filtration rate (79 mL/min/1.73m ^2^ ) were evident. Furthermore, a marked elevation in erythrocyte sedimentation rate (93 mm/hr) was identified, indicating a significant inflammatory condition.

**Fig. 3:**
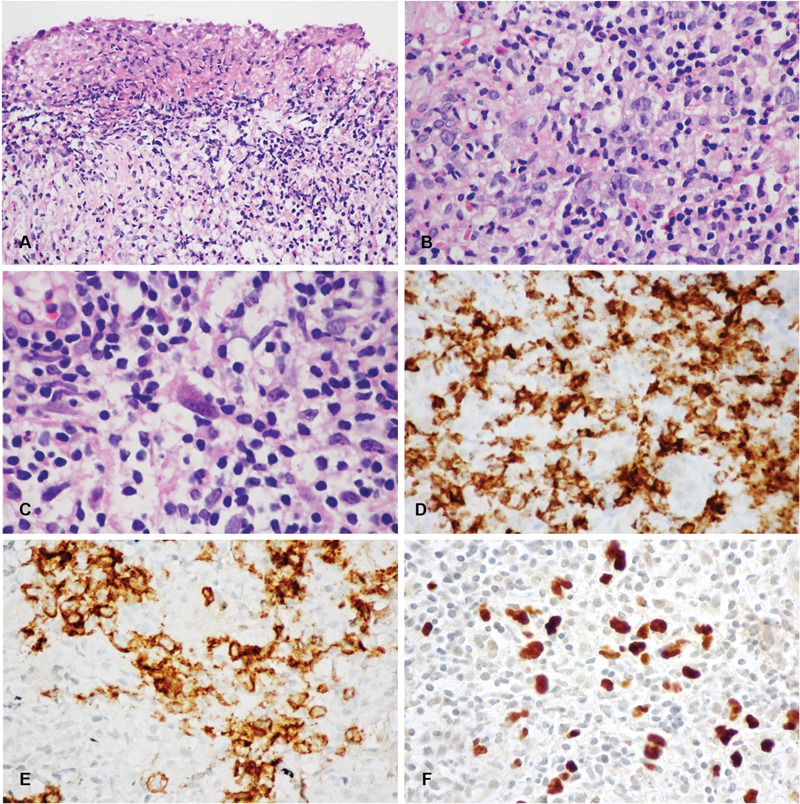
Histopathologic features and immunophenotypic profile of Epstein–Barr virus-positive mucocutaneous ulcer. ( **A** ) Surface ulceration covered by fibrinous exudate (hematoxylin and eosin [H&E], x 200). ( **B** ) Proliferation of atypical lymphoid cells intermingled with mixed inflammatory infiltrate (H&E, x 400). ( **C** ) Occasional Hodgkin and Reed/Sternberg-like cells (H&E, x 600). ( **D** ) Atypical lymphoid cells showing diffuse and strong expression of CD20. ( **E** ) CD30 and ( **F** ) Epstein–Barr virus-encoded small RNA *in situ* hybridization showing Epstein–Barr virus -infected cells within the lesion (x 400).

The patient had a previous biopsy submitted elsewhere 1 month prior and was diagnosed as a nonspecific ulcer with chronic inflammation. The present specimen was taken from the right palatal area. Histopathologic examination revealed an extensive ulceration covered with fibrinous exudate ( Fig. 3A ). The underlying connective tissue demonstrated diffuse, intense polymorphous inflammatory infiltrate, consisting of scattered atypical lymphoid cells of varying sizes admixed with numerous lymphocytes, plasma cells, histiocytes, neutrophils, and scattered eosinophils ( Fig. 3B ). Occasional immunoblasts are also noted. Some atypical cells displayed Hodgkin and Reed/Sternberg (HRS)-like cell features within the small lymphocytic background ( Fig. 3C ).

Further immunohistochemical and *in situ* hybridization (ISH) investigations were performed. Atypical lymphoid cells including HRS-like cells displayed diffuse and strong immunoreactivity for CD 20 ( Fig. 3D ) and IRF4/MUM1, representing the transformed and activated B cell phenotype. Additionally, CD 30 was expressed by scattered atypical cells ( Fig. 3E ). CD 15 was nonreactive in atypical cells but highlighted neutrophils. CD 3 stained the background T lymphocytes. Epstein–Barr virus (EBV)-encoded small RNA revealed positive staining in the nuclei of lesional cells ( Fig. 3F ). Based on the patient history, clinical and histopathologic features as well as the immunophenotyping profile, the diagnosis of EBVMCU was rendered.

**Fig. 4:**
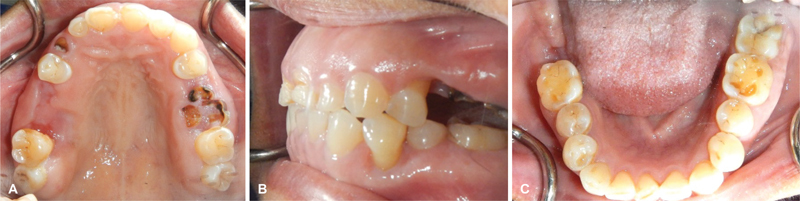
Two months following the cessation of both methotrexate and leflunomide ( **A–C** ), all lesions show spontaneous regression.

The patient was then referred to her rheumatologist for possible immunosuppressive drug discontinuation or dose adjustment. Two weeks following the cessation of both MTX and leflunomide, the ulcers were partially re-epithelialized. At 2-month follow-up, all lesions nearly showed complete regression ( Fig. 4A-C ). Although the patient reported a markedly decreased oral pain, she had experienced flares of RA symptoms. Thus, her rheumatologist considered the resumption of low-dose MTX (an initial dose of 5 mg twice weekly, and gradually increased to 10 mg twice weekly) in combination with sulfasalazine (1,000 mg daily). At 9-month follow-up, no evidence of relapsing oral EBVMCU was observed.

## Discussion

EBVMCU is a relatively new disease entity, first included in the 2017 WHO classification of tumors of hematopoietic and lymphoid tissues. 2 Clinically, oral EBVMCUs most frequently arise in gingiva, tongue, and palate, but can occur in any intraoral sites, including lip, buccal mucosa, and floor of the mouth. 1 5 7 8 The recurrent site of oral EBVMCU may reflect the initial site of EBV inoculation in this region. Multifocal EBVMCUs as in the present case were uncommon, representing only 10 to 17% of documented cases. 5 8 The extensive form of EBVMCU may possibly reflect a more profound decrease in immunosurveillance, triggered by the long-term use of both MTX and leflunomide.

The dual drug combination is widely used to improve the treatment efficacy in active RA patients who failed the first-line drug therapy, such as MTX. MTX is a folic acid analog that binds irreversibly to dihydrofolate reductase, and functions as a folic acid antagonist. It exerts the antiproliferative property by targeting critical folate-dependent enzymatic steps in the de novo synthesis of purines and pyrimidines, the building blocks for RNA and DNA synthesis. 9 This drug usually affects rapidly renewing tissues, such as bone marrow or gastrointestinal mucosa. Several adverse reactions from long-term low-dose MTX treatment have been reported, including myelosuppression, gastrointestinal and mucocutaneous toxicity, hepatotoxicity, and acute kidney failure. 10 Oral ulcerations can occur as an acute mucocutaneous toxicity of MTX, accounting for 11 to 17% of patients using this drug. 10 This may be related to the elevated MTX plasma level, MTX overdose, or folic acid deficiency. 11 12 These lesions are often seen as shallow ulcers, 8 exhibiting an acute onset with rapid progression and variable healing time after MTX discontinuation. 10 11 12 In contrast, oral EBVMCUs are sharply demarcated, deep ulcer and has an indolent clinical course.

While MTX is known to be associated with EBVMCU in a subgroup of immunosuppressed patients due to iatrogenic causes, 8 leflunomide as an additional causative agent has not been previously reported in the literature. Leflunomide is an immunomodulatory and immunosuppressive agent that inhibits pyrimidine synthesis and interferes with cell cycle progression, resulting in decreased T cell proliferation. The known adverse reactions of leflunomide therapy include gastrointestinal side effects, hepatotoxicity, cutaneous ulcers, 13 and oral complications, such as candidiasis, herpetic infection, or nonspecific ulcers. 14 15

Owing to the patient history and presentation of multiple long-standing deep ulcers of varying sizes, the clinical differential diagnostic list of our case spans a diverse type of oral ulcerations, including traumatic ulcerative granuloma with eosinophilia (TUGSE), deep fungal infections, tuberculosis (TB), necrotizing ulcerative gingivitis (NUG), EBVMCU, and other LPDs.

TUGSE is a unique type of chronic ulceration, of which local trauma may be a major contributing factor. 16 Nevertheless, many cases present without existing evidence of trauma. Clinically, TUGSE appears as an isolated mucosal ulcer or mass often with a rolled hyperkeratotic collar, most often affecting the tongue. It can also affect other intraoral sites, such as gingiva, buccal mucosa, palate, and lip. Multifocal form of TUGSE is unusual, but has also been reported in the literature. 17 TUGSE is a self-limiting condition that can persist for several weeks or months and slowly resolves without any treatment. 16 Notably, many of these lesions rapidly undergo resolution following their biopsy. 18 In contrast to our cases, the lesions persisted even after the previous incisional biopsy.

Oral TB most often represents a secondary infection from the initial pulmonary foci. 19 Likewise, oral deep fungal infections usually manifest along with the disseminated form of diseases. These infections mainly occur in immunocompromised settings and frequently appear as chronic oral ulcerations with surface irregularity similar to EBVMCU. However, patients with TB or deep fungal infections usually have systemic and respiratory signs and symptoms. Thus, correlation with patient history, clinical findings, chest radiographs, and additional microbial investigations is helpful to separate these infections from EBVMCU.

NUG is a distinctive pathologic condition, associated with spirochete and fusobacterium infections. It may occur in any age group, but most often affects adolescents. 20 It frequently arises in immunocompromised patients, especially in HIV-infected patients. 21 NUG clinically exhibits a rapid onset of characteristic punched-out, crater-like necrosis of the interdental papillae, associated with gingival pain, bleeding, and halitosis. In contrast to EBVMCU, systemic symptoms, such as lymphadenopathy and malaise, are common. In addition, NUG demonstrates rapid resolution after removal of bacterial challenge by periodontal debridement, scaling, and curettage as well as additional chlorhexidine or diluted hydrogen peroxide mouth rinse.

EBVMCU and oral CD30-positive lymphoproliferative disorder (CD30+ LPD) may be clinically indistinguishable and further histopathologic and immunohistochemical studies are required to obtain the definite diagnosis. Microscopically, EBVMCUs can show a wide spectrum of histopathologic features. A recent study divided EBVMCUs into four morphologic subtypes, the polymorphous, large cell-rich, classic Hodgkin lymphoma (CHL)-like, and mucosa-associated lymphoid tissue lymphoma-like. 7 The present case microscopically displayed polymorphic inflammatory infiltrate admixed with various small-to-large atypical EBV-positive lymphoid cells with scattered atypical lymphoid cells and occasional HRS-like cells. Such features are consistent with the reportedly most prevalent polymorphous subtype.

The most common microscopic differential diagnoses of EBVMCU are other entities of LPDs, including CHL, diffuse large B cell lymphoma (DLBCL), or CD30+ LPD. In addition, one of the more common oral ulcerations, such as TUGSE, may show some histopathologic resemblance to EBVMCU. CHL shares similar histomorphologic features with EBVMCU in that both are composed of variable numbers of atypical lymphoid cells with HRS cell morphology admixed with a rich, polymorphic inflammatory background. However, CHL is mostly a nodal disease and the extranodal involvement is exceedingly rare. 8 22 EBVMCU and CHL can also be distinguished based on the immunophenotype. Large HRS-like cells and immunoblasts in EBVMCU are CD45 positive, whereas neoplastic cells in CHL lack the expression of this protein. 1 4 In addition, EBVMCUs often have abundant CD20-positive atypical B lymphocytes, while only a minority of tumor cells in CHL express this marker. 1 4 The prototypic Reed/Sternberg (RS) cells in CHL co-express both CD30 and CD15, whereas HRS-like cells in EBVMCU express CD30, with limited CD15 positivity in approximately half of the cases. 1 22 Besides, both markers often highlight a wide range of EBV-positive cells of varying sizes in EBVMCU; however, in CHL, their expression is restricted to the neoplastic RS cells only. 4 Lastly, while HRS-cells of CHL typically lack the transcription factors OCT2 and BOB1 and retain weak expression of PAX5, the atypical cells of EBVMCU are often positive for PAX5, and OCT2, with variable expression of BOB1. 1

Similar to EBVMCU, large atypical EBV-positive lymphoid cells are characteristics of EBV-positive DLBCL, the high-grade B cell neoplasm, generally occurring in the elderly. 3 22 Its neoplastic component is composed of a variable number of large, transformed cells/immunoblasts, and HRS-like cells, within the reactive mixed inflammatory infiltrate. 23 These tumor cells demonstrate positive immunoreactivity for CD20, CD30, and sometimes CD15, 22 and show an activated-B cell immunophenotype, being positive for IRF4/MUM1 and negative for CD10 and BCL6. 23 These findings are distinctly resembling EBVMCU. However, microscopically EBVMCU exhibits a sharply circumscribed base and the atypical B cells are limited to the base of the ulcer, with minimal extension into the deep connective tissue. 24 In contrast, the diffusely infiltrative nature of tumor cells is omnipresent in EBV-positive DLBCL. In addition, EBVMCU clinically differs from EBV-positive DLBCL in that it presents as a localized lesion without systemic involvement. 22

A few studies investigated the immunoglobulin heavy chain (IGH) and T cell receptor (TCR) rearrangement in EBVMCU and EBV-positive DLBCL. The 39% clonal IG gene rearrangement with 38% clonal and 31% restricted T cell patterns was reported in EBVMCUs, suggesting a reactive B cell proliferation with limited T cell repertoire to EBV. 1 These features are in contrast to those of DLBCLs, which showed a relatively higher frequency of clonal IGH rearrangements 7 and lower frequency of clonal TCR rearrangement. 3 However, these findings were not statistically significant and further studies are required to potentially establish these differences for future diagnostic use. The summary of histopathologic and immunophenotypic features of EBVMCU and other LPDs is presented in Table 1 .

**Table 1:** The histopathologic characteristics and immunophenotypes of EBVMCU and its mimics

Entities	Histopathologic features	Immunophenotypes	Clonal IG gene rearrangements
EBVMCU	Well-circumscribed ulcer Large, transformed cells/immunoblasts, and HRS-like cells Polymorphic inflammatory background, scattered apoptotic cells Band-like infiltrate of mature T lymphocytes at the ulcer base	CD20 + , CD45 + , CD30 + , CD15−/+ , IRF4/MUM1 + , PAX5 + , OCT2 + , BOB1 +/− , EBER+	Fewer than 50% of cases
CHL	Effaced nodal architecture Variable numbers of atypical HRS cells Polymorphic inflammatory background	CD20 −/+ a , CD45−, CD30 + , CD15 + , IRF4/MUM1 + , PAX5 weak + , OCT2−, BOB1− , EBER+	More than 98% of cases
EBV-positive DLBCL	Variable number of large, transformed cells/immunoblasts and HRS-like cells, Polymorphic or monomorphic pattern Geographical necrosis and angioinvasion may be present	CD20 + , CD45 + , CD30 + , CD15−/+ , CD10−, IRF4/MUM1 + , PAX5 + , OCT2 + , BOB1 + , EBER+	Clonality of the IG genes can usually be detected b
CD30+ LPD	Ulcer with an infiltrate of large, atypical CD30+ T cells in a polymorphic inflammatory background	T cell markers+ (CD2 + , CD3 + , CD4 + ), CD30 + , EBER−	Not detected (Monoclonal TCR gene rearrangement present)

Abbreviations: EBVMCU, Epstein–Barr virus-positive mucocutaneous ulcer; IG, immunoglobulin; HRS, Hodgkin/Reed-Sternberg; CHL, classic Hodgkin lymphoma; EBV-positive DLBCL, EBV-positive diffuse large B cell lymphoma; CD30+ LPD, CD30-positive T cell lymphoproliferative disorder; TCR, T cell receptor.

a Usually expressed on a minority of neoplastic cells.

b No significant difference between EBVMCU and EBV-positive DLBCL.

The presence of dense lymphoid infiltrates, composed of CD30+ atypical cells with a polymorphous infiltrate in the background, as well as vasculitic changes as in the present case may be reminiscent of CD30+ LPD or TUGSE. However, in both lesions, these cells exhibit predominantly T cell phenotype, 25 in contrast to the B phenotype identified in EBVMCU. 1 4 In addition, the mixed inflammatory infiltrate in TUGSE often extends deeper into the submucosa causing degeneration of underlying muscle fibers, 16 while EBVMCU exhibits a well-circumscribed base with a band-like infiltrate of mature T lymphocytes at the deepest margin. 24

Distinguishing EBVMCU from its mimics histopathologically is necessary, because EBVMCU exhibits an indolent clinical behavior with better prognosis than other EBV-positive LPDs and rarely requires intensive treatment. Even though no evidence-based guideline for managing EBVMCU has been established to date, the majority of EBVMCU patients exhibit complete or partial remission after the cessation or modification of the immunosuppressive treatment. In some recalcitrant cases, further managements, including rituximab therapy, surgical excision, R-CHOP chemotherapy, or radiation therapy, may be required to achieve remission. 3

## Conclusion

EBVMCU is a rare, unique entity of LPDs with overlapping clinical and histopathological features with other benign and malignant lesions. Therefore, the EBVMCU diagnosis requires comprehensive correlation between patient history, clinical findings, and histopathological features including additional immunohistochemical and ISH investigations. The multifocal form of EBVMCU as in the present case is uncommon and could reflect the intensified immunosuppression from the dual MTX and leflunomide therapy. Because of its benign nature and evidently better prognosis than most of its potential clinical and histopathologic mimics, accurately diagnosing EBVMCU can help avoid unnecessary patient management and achieve optimal outcomes.
